# Mutations in the voltage‐sensing domain affect the alternative ion permeation pathway in the TRPM3 channel

**DOI:** 10.1113/JP274124

**Published:** 2018-04-25

**Authors:** Katharina Held, Fabian Gruss, Vincenzo Davide Aloi, Annelies Janssens, Chris Ulens, Thomas Voets, Joris Vriens

**Affiliations:** ^1^ Laboratory of Experimental Gynecology and G‐PURE, KU Leuven Department of Development and Regeneration Herestraat 49 box 611 B‐3000 Leuven Belgium; ^2^ Laboratory of Ion Channel Research and TRP Research Platform Leuven (TRPLe), KU Leuven Department of Cellular and Molecular Medicine Herestraat 49 box 802 B‐3000 Leuven Belgium; ^3^ VIB Center for Brain & Disease Research B‐3000 Leuven Belgium; ^4^ Laboratory of Structural Neurobiology and TRP Research Platform Leuven (TRPLe), KU Leuven Department of Cellular and Molecular Medicine Herestraat 49 box 601 B‐3000 Leuven Belgium

**Keywords:** TRP, TRPM3, alternative ion permeation pathway

## Abstract

**Key points:**

- Mutagenesis at positively charged amino acids (arginines and lysines) (R1–R4) in the voltage‐sensor domain (transmembrane segment (S) 4) of voltage‐gated Na^+^, K^+^ and Ca^2+^ channels can lead to an alternative ion permeation pathway distinct from the central pore.
- Recently, a non‐canonical ion permeation pathway was described in TRPM3, a member of the transient receptor potential (TRP) superfamily. The non‐canonical pore exists in the native TRPM3 channel and can be activated by co‐stimulation of the endogenous agonist pregnenolone sulphate and the antifungal drug clotrimazole or by stimulation of the synthetic agonist CIM0216.
- Alignment of the voltage sensor of Shaker K^+^ channels with the entire TRPM3 sequence revealed the highest degree of similarity in the putative S4 region of TRPM3, and suggested that only one single gating charge arginine (R2) in the putative S4 region is conserved.
- Mutagenesis studies in the voltage‐sensing domain of TRPM3 revealed several residues in the voltage sensor (S4) as well as in S1 and S3 that are crucial for the occurrence of the non‐canonical inward currents.
- In conclusion, this study provides evidence for the involvement of the voltage‐sensing domain of TRPM3 in the formation of an alternative ion permeation pathway.

**Abstract:**

Transient receptor potential (TRP) channels are cationic channels involved in a broad array of functions, including homeostasis, motility and sensory functions. TRP channel subunits consist of six transmembrane segments (S1–S6), and form tetrameric channels with a central pore formed by the region encompassing S5 and S6. Recently, evidence was provided for the existence of an alternative ion permeation pathway in TRPM3, which allows large inward currents upon hyperpolarization independently of the central pore. However, very little knowledge is available concerning the localization of this alternative pathway in the native TRPM3 channel protein. Guided by sequence homology with Shaker K^+^ channels, in which mutations in S4 can create an analogous ‘omega’ pore, we performed site‐directed mutagenesis studies and patch clamp experiments to identify amino acid residues involved in the formation of the non‐canonical pore in TRPM3. Based on our results, we pinpoint four residues in S4 (W982, R985, D988 and G991) as crucial determinants of the properties of the alternative ion permeation pathway.

## Key points

- Mutagenesis at positively charged amino acids (arginines and lysines) (R1–R4) in the voltage‐sensor domain (transmembrane segment (S) 4) of voltage‐gated Na^+^, K^+^ and Ca^2+^ channels can lead to an alternative ion permeation pathway distinct from the central pore.
- Recently, a non‐canonical ion permeation pathway was described in TRPM3, a member of the transient receptor potential (TRP) superfamily. The non‐canonical pore exists in the native TRPM3 channel and can be activated by co‐stimulation of the endogenous agonist pregnenolone sulphate and the antifungal drug clotrimazole or by stimulation of the synthetic agonist CIM0216.
- Alignment of the voltage sensor of Shaker K^+^ channels with the entire TRPM3 sequence revealed the highest degree of similarity in the putative S4 region of TRPM3, and suggested that only one single gating charge arginine (R2) in the putative S4 region is conserved.
- Mutagenesis studies in the voltage‐sensing domain of TRPM3 revealed several residues in the voltage sensor (S4) as well as in S1 and S3 that are crucial for the occurrence of the non‐canonical inward currents.
- In conclusion, this study provides evidence for the involvement of the voltage‐sensing domain of TRPM3 in the formation of an alternative ion permeation pathway.

## Introduction

Voltage‐gated cation channels are classically described as membrane proteins with a single, central aqueous pore, regulating the flow of cations such as Na^+^, K^+^ and Ca^2+^. In general, voltage‐gated cation channels consist out of two modules, the voltage‐sensing domain (VSD) formed by transmembrane segments S1–S4, and the pore domain formed by segments S5, S6 and the pore loop. The VSD enables these channels to detect and react to changes in voltages (Bezanilla, 2000; Horn, 2002; Catterall, 2010). An important region of the VSD is transmembrane segment 4 (S4), as it carries a number of positively charged arginines (R) and lysines (K) that underlie voltage sensitivity (Bezanilla, 2000; Catterall, 2010). Upon depolarization of the cell membrane, S4 movement will occur and accomplish the transport of the positive S4 charges across the membrane electric field (Bezanilla, 2002). This causes a conformational change of the channel and results in opening of the central pore (Horn, 2002; Catterall, 2010). In recent years, evidence has accumulated for the existence of an additional ion permeation pathway, distinct from the central pore. The presence of a ‘non‐canonical’ ion permeation pathway was first described in Shaker K^+^ channels, in which neutralization of the first gating charge in S4 allowed the permeation of protons or metal cations through an ionic gap in the VSD of the channel (Starace & Bezanilla, 2004; Tombola *et al*. 2005). Subsequently, similar non‐canonical pores were described in disease‐causing mutants of voltage‐gated K^+^, Na^+^ and Ca^2+^ channels (Sokolov *et al*. 2007; Gosselin‐Badaroudine *et al*. 2012; Miceli *et al*. 2012; Wu *et al*. 2012), indicating the pathophysiological relevance of non‐canonical ion permeation pathways (reviewed in Held *et al*. 2016). Recently, we have shown the presence of a non‐canonical pore in TRPM3 (Vriens *et al*. 2014), a member of the transient receptor potential (TRP) superfamily, which shows a similar architecture to voltage‐gated K^+^, Ca^2+^ and Na^+^ channels. TRPM3 is an example of a polymodally gated ion channel, as it can be activated by a plethora of stimuli including voltage, chemicals such as pregnenolone sulphate (PS), nifedipine and CIM0216, and physical stimuli such as temperature and hypotonicity‐induced cell swelling (Grimm *et al*. 2003; Wagner *et al*. 2008; Vriens *et al*. 2011; Held *et al*. 2015). Activation of TRPM3 by PS results in an outwardly rectifying current (*I*)–voltage (*V*) relationship. However, combined application of PS and the antifungal drug clotrimazole (Clt) or sole application of CIM0216 induces currents with an *I*–*V* relationship that rectifies in both inward and outward directions. The appearance of the inward rectifying currents at negative voltages could be attributed to the opening of a non‐canonical pore in TRPM3 (Vriens *et al*. 2014; Held *et al*. 2015). This non‐canonical ion permeation pathway differs from the central pore with respect to its rectification, a lower permeability to Ca^2+^ and Mg^2+^, resistance to Ca^2+^‐dependent desensitization and insensitivity to the open pore blocker La^3+^ (Vriens *et al*. 2014; Held *et al*. 2015). Interestingly, in contrast to voltage‐gated Na^+^, K^+^ and Ca^2+^ channels, the non‐canonical ionic current in TRPM3 occurs in the wild‐type channel, and depends on the presence of specific ligands or ligand combinations. At the moment, little is known about the domains of the channel protein that constitute the non‐canonical pore in TRPM3. Based on site‐directed mutagenesis and homology with the Shaker K^+^ channel, we here provide evidence for a key role of the S4 domain in mediating the currents through the non‐canonical pore in TRPM3.

## Methods

### Cell culture

HEK293 cells stably expressing murine TRPM3 (HEK‐TRPM3) were designed and cultured as described previously (Vriens *et al*. 2011). HEK293 cells were transiently transfected with 2 μg of DNA using TransIT transfection reagent (Mirus Bio, Madison, WI, USA) 24–72 h before the measurements.

### Site‐directed mutagenesis

All mutants were obtained by the standard PCR overlap extension method using mTRPM3α2 from pCAGGS/IRES‐GFP vector (Vriens *et al*. 2007). Accuracy of all mutant sequences was verified by sequencing of all DNA constructs.

### Electrophysiology and fluorescence imaging

During fractional Ca^2+^ current experiments, cells were illuminated at 380 nm, monitoring [Ca^2+^]_cyt_ from Fura‐K^+^ loaded cells using a monochromator‐based system consisting of a Polychrome IV monochromator (FEI Munich GmbH, Munich, Germany) with an additional TILL photonics photomultiplier, both controlled by Pulse software (HEKA Elektronik, Lambrecht, Germany). Standard intracellular solution contained (in mm): 100 CsAsp, 45 CsCl, 10 Hepes, 1 MgCl_2_ and 5 K_5_Fura‐2 (pH 7.2 with CsOH). The extracellular solutions contained (in mm): 10 Hepes and 1 MgCl_2_ with added 150 NaCl and 1 EGTA for the Ca^2+^‐free solution and 150 NaCl and 2 CaCl_2_ for the 2 Ca^2+^ solution (pH 7.4 with NaOH). The isotonic Ca^2+^ solution contained (in mm): 100 CaCl_2_ and 10 Hepes (pH 7.4 with Ca(OH)_2_). Before the measurements, cells were dialysed for up to 5 min with a patch pipette containing the intracellular solution allowing the cell to load with K_5_Fura‐2. The current fraction carried by Ca^2+^ (*F*%) was then determined as:

$$F\%\;=\;100\times \;\frac{\int I_{100}dt}{\int I_{2}dt}\times \;\frac{\Delta \;F_{380,2}}{\Delta \;F_{380,100}}$$

 where *I*_2_ and *I*_100_ represent the current at −80 mV in 2 mm and 100 mm Ca^2+^, respectively, and Δ*F*_380,2_ and Δ*F*_380,100_ the corresponding decreases in Fura‐2 fluorescence at 380 nm. The ratio of Δ*F*_380,100_ and $\int I_{100}dt$ describes the situation where 100% of the permeating ion is carried by Ca^2+^. Therefore, the ratio of Δ*F*_380,2_ and $\int I_{2}dt$ was normalized to the ratio of Δ*F*_380,100_ and $\int I_{100}dt$ to determine the fraction of Ca^2+^ permeating the pore in a standard bath solution of 2 mm Ca^2+^.

Standard patch clamp recordings were made with an EPC‐10 amplifier and the PatchMasterPro software (HEKA Elektronik). Current measurements were performed at a sampling rate of 20 kHz and currents were digitally filtered at 2.9 kHz. In all measurements, 70% of the series resistance was compensated.

For whole‐cell recordings on HEK293 cells, the standard internal solution contained (in mm): 100 CsAsp, 45 CsCl, 10 EGTA, 10 Hepes, 1 MgCl_2_ (pH 7.2 with CsOH); and the standard extracellular solution contained (in mm): 150 NaCl, 1 MgCl_2_, 10 Hepes (pH 7.4 with NaOH). The standard patch pipette resistance was between 2 and 4 MΩ when filled with pipette solution.

Single‐channel measurements were performed in the on‐cell configuration of the patch clamp technique. The extracellular solution contained (in mm): 150 KCl, 1 MgCl_2_, 10 Hepes (pH 7.4 with KOH); and the pipette solution contained (in mm): 150 NaCl, 1 MgCl_2_, 10 Hepes (pH 7.4 with NaOH).

### Statistics

Electrophysiological data were analysed using IgorPro 6.2 (WaveMetrics, Lake Oswego, OR, USA), WinASCD (Guy Droogmans, Leuven) and OriginPro 8.6 (OriginLab Corp., Northampton, MA, USA). OriginPro 8.6 was further used for statistical analysis and data display. All data sets were tested for normality and Student's paired, two‐tailed *t* test or the Mann–Whitney test was used for statistical comparison between different data sets. *P* values below 0.05 were considered as significantly different. Data points represent means ± SEM of the given number (*n*) of identical experiments.

Conductance–voltage (*G–V*) curves were fitted with a Boltzmann function of the form:

$$G\left(V\right)=\frac{G_{\max}}{1+\exp\left(zF\frac{\left(V_{1/2}-V\right)}{RT}\right)},$$

where *z* is the apparent gating charge, *V*_1/2_ the potential for half‐maximal activation, *G*_max_ the maximal conductance, *F* the Faraday constant, *R* the gas constant and *T* the absolute temperature. Experiments were performed at room temperature.

Probability density function histograms were generated from single‐channel traces that were low‐pass filtered in WinASCD prior to analyses. This allowed better visualization of the low frequency small conductance openings that are characteristic for the alternative ion permeation pathway, while blunting the high frequency signals characteristic for the central pore openings of TRPM3 (Vriens *et al*. 2014).

### Homology modelling

A homology model of the transmembrane region of mTRPM3α2 (residues 862–1235) was generated using SWISS‐MODEL server (Biasini *et al*. 2014). As template, a TRPM4 cryo‐EM structure (PDB: 6bco; Guo *et al*. 2017) was used that has 31% sequence identity to TRPM3 in the modelled region. The generated model has a QMEAN score of −3.67, indicating acceptable overall quality (Benkert *et al*. 2011).

## Results

### Positive charge insertion at W982 in TRPM3 at the position corresponding to R1 inhibits the non‐canonical pore currents

**Figure 1:**
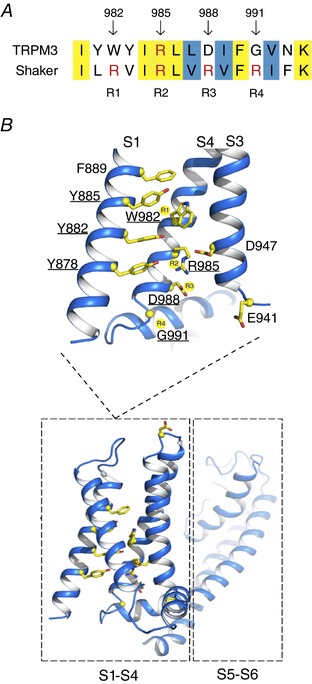
Properties of the TRPM3 VSD *A*, alignment of transmembrane segment 4 (S4) of TRPM3 with the Shaker K^+^ channel. R1–R4 indicate the four different gating charge positions as described in the Shaker channel (Aggarwal & MacKinnon, 1996). Yellow background indicates matched amino acids, blue background indicates amino acids belonging to similar subgroups. *B*, homology model of the transmembrane region of TRPM3 (based on the cryo‐EM structure of TRPM4 (PDB: 6bco; Guo *et al*. 2017) depicting S1–S6 and indicating the four gating charge positions (R1–R4) in S4 as shown in *A*. Residues investigated in this study are shown in yellow stick representation. Residues functionally important in mediating the alternative current in TRPM3 are underlined.

**Figure 2:**
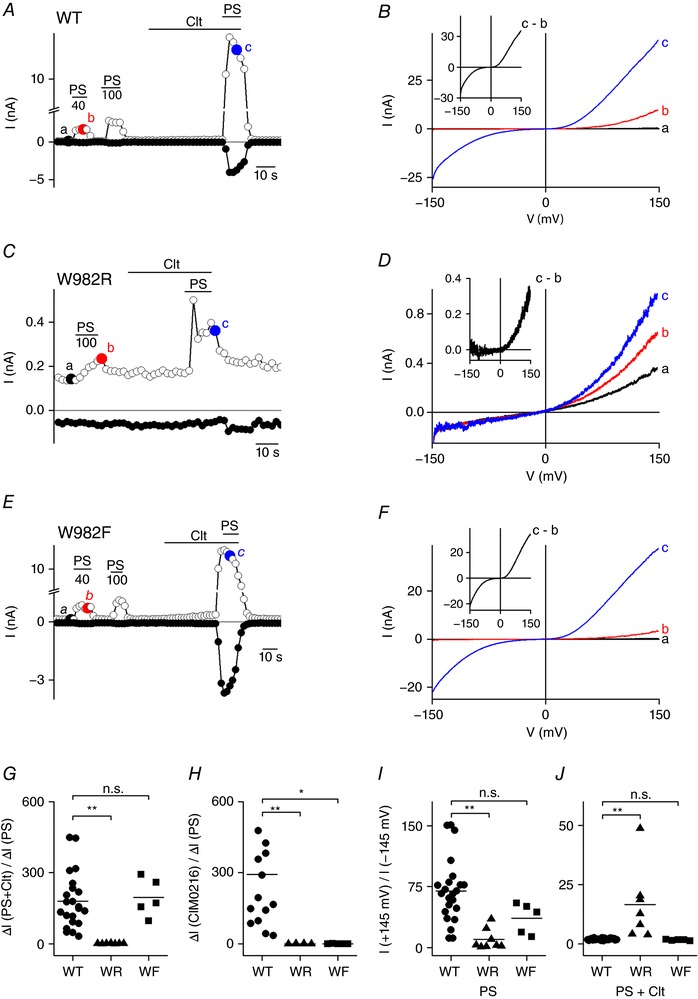
An arginine at position R1 abolishes the Clt+PS‐induced inward currents *A*, time course of whole‐cell currents of HEK‐TRPM3 cells at ±80 mV upon application of PS (40, 100 μm), Clt (10 μm) or PS+Clt (with PS 40 μm). *B*, *I–V* relationship of time points indicated in *A*. Inset, Clt‐induced current increase obtained as the difference between two traces in *B*. *C*, similar to *A*, HEK293 cells transiently overexpressing W982R upon application of PS (100 μm) and Clt (10 μm). *D*, *I–V* relationship of time points indicated in *C*. *E*, as in *A* and *C*, but in HEK293 cells transiently expressing the TRPM3 mutant W982F. *F*, *I–V* relationship of time points indicated in *E*. *G*, *x*‐fold current increase at −145 mV upon co‐application of PS+Clt normalized to the *x*‐fold increase upon single application of PS in WT‐TRPM3 and the mutants W982R (WR) and W982F (WF). *H* similar to *G*, but for CIM0216 application normalized to PS. *I*, PS‐induced rectification defined by the current at +145 mV divided by the current at −145 mV in native TRPM3 (WT) and indicated mutants. *J*, Clt+PS‐induced rectification defined by the current at +145 mV divided by the current at −145 mV in TRPM3 (WT) and indicated mutants; Mann–Whitney test was used for statistical calculations and *P* values were corrected with the Bonferroni correction *P*/*n* with *n* = number of mutants; ^*^<2.9 × 10^−3^, ^**^<5.9 × 10^−4^, ^***^<5.9 × 10^−5^; n.s., not significant.

In voltage‐gated K^+^, Na^+^ and Ca^2+^ channels, the non‐canonical pore is not present in the wild‐type channel, and can only be uncovered by specific mutations in the VSD. For instance, mutating the outermost arginine in transmembrane segment 4 (S4) of the Shaker K^+^ channel to smaller, uncharged residues induces the appearance of a non‐canonical current at negative membrane potentials. This indicates the existence of an ion‐conducting pore in the VSD that is normally occluded by the arginine residue (Tombola *et al*. 2005). The S4 of the VSD in voltage‐dependent ion channels carries a number of positively charged arginine or lysine residues, normally at every third amino acid position (Bezanilla, 2000; Catterall, 2010). Alignment of a 15‐amino‐acid region encompassing the voltage‐sensing arginines of the Shaker K^+^ channel with the entire TRPM3 sequence revealed a high degree of similarity in the putative S4 region of TRPM3 (40% identity, 60% similarity) (Fig. 1 *A*). This alignment indicates that only a single gating charge arginine is conserved in the putative S4 of TRPM3, namely at the position corresponding to the second arginine (R2) of the Shaker K^+^ channel. The positions corresponding to R1, R3 and R4 in Shaker feature other, non‐positively charged amino acids in TRPM3 (Fig. 1 *A* and *B*). In previous work, it was shown that introducing an arginine in TRPM3 at the position corresponding to R1 of the Shaker K^+^ channel (W982R) specifically inhibits currents through the non‐canonical pore (Vriens *et al*. 2014; Held *et al*. 2015). The hallmark feature of TRPM3 currents in the presence of Clt and PS is the double rectification pattern, with marked inward currents at strongly hyperpolarizing potentials, compared to the strictly outwardly rectifying PS‐activated currents in the absence of Clt (Fig. 2 *A* and *B* plus inset, and Table 1). The difference in *I*–*V* relations can be quantified from the rectification score, which was determined as the ratio of absolute current amplitude at +145 mV and −145 mV (rectification_+145/−145_). For PS‐induced currents, rectification_+145/−145_ amounted to 69.74 ± 8.20 compared to 1.89 ± 0.08 in the combined presence of Clt and PS in wild‐type (*n* = 23) (Fig. 2 *I* and *J*). Mutating tryptophan to arginine at position 982, a position equivalent to R1, resulted in a mutant channel (W982R) that was expressed at lower levels than wild‐type TRPM3. Interestingly, co‐application of Clt and PS to the W982R mutant failed to induce an inwardly rectifying current component, resulting in similar rectification scores for the PS‐activated current in the absence and presence of Clt (Fig. 2 *C*, *D*, *G*, *I* and *J*, and Table 1). Similarly, application of CIM0216 also resulted in an outwardly rectifying *I–V* relationship leading to equivalent increases in inward current amplitudes as in PS and PS+Clt conditions (Fig. 2 *H* and Table 2), in line with previous reports (Vriens *et al*. 2014; Held *et al*. 2015). In contrast, mutating the tryptophan to a more conservative phenylalanine (W982F), resulted in a mutant channel in which the inward current amplitudes and current rectification of PS‐activated currents in the absence or presence of Clt were similar to wild‐type (Fig. 2 *E*–*G*, *I* and *J*, and Table 1). Interestingly, CIM0216 stimulation did not induce any current increase in W982F‐transfected HEK293 cells (Fig. 2 *H* and Table 2).

**Table 1:** Comparison of PS‐ and Clt+PS‐induced activation of native TRPM3 and mutants

Channel	Segment	Δ*I*_−145mV_ PS (pA)	Δ*I*_−145mV_ PS+Clt (pA)	Ratio Δ*I* (PS+Clt)/Δ*I* (PS)	Significance
NT	–	NA	NA	–	–
WT	–	−44.7 ± 9.0	−5697.2 ± 907.6	180.0 ± 25.0	–
W982R^†^	S4	−9.2 ± 3.5	−7.5 ± 2.0	0.8 ± 0.3	**
W982F	S4	−87.6 ± 36.6	−14743.4 ± 2997.6	194.9 ± 35.7	0.51
R985A	S4	−3.3 ± 0.9	−3.8 ± 2.5	1.2 ± 0.6	*
R985Q	S4	−15.0 ± 5.6	−29.9 ± 15.5	1.5 ± 0.3	***
D988R	S4	−77.3 ± 27.9	−243.4 ± 110.4	2.1 ± 0.6	*
D988K	S4	−3.3 ± 1.0	−30.8 ± 13.3	17.2 ± 7.8	***
D988E	S4	−198.2 ± 70.6	−11337.6 ± 2101.4	202.9 ± 83.2	0.26
G991R	S4	−328.6 ± 91.0	−2965.6 ± 806.3	10.6 ± 2.6	**
G991A	S4	−184.4 ± 85.7	−3896.4 ± 1688.7	26.8 ± 6.6	**
Y981L	S4	−133.0 ± 31.0	−46.5 ± 8.3	0.4 ± 0.1	**
Y983V	S4	NA	NA	–	–
N993F	S4	−19.6 ± 10.0	−207.0 ± 36.9	20.9 ± 7.1	*
Y878T	S1	−99.7 ± 55.9	−115.6 ± 66.8	1.2 ± 0.2	*
Y882T	S1	−3752.6 ± 493.1	−3853.9 ± 401.4	1.0 ± 0.1	*
Y885T	S1	−87.2 ± 23.9	−316.8 ± 89.4	3.6 ± 0.1	*
F889L	S1	−12.4 ± 8.6	−262.6 ± 72.7	64.3 ± 23.3	0.027
E941Q	S3	−62.7 ± 20.9	−1573.6 ± 351.9	34.7 ± 7.5	**
D947N	S3	NA	NA	–	–
D964N	S3	−272.6 ± 51.0	−6315.5 ± 1060.2	24.4 ± 2.4	***
D947R R985D	S3	NA	NA	–	–

The delta current increase at −145 mV (Δ*I*_−145mV_) induced by PS (40 μm) Δ*I* (PS) or by co‐application of Clt (10 μm) + PS (40 μm) Δ*I* (PS+Clt) and the ratio of the *x*‐fold current increases calculated as Δ*I* (PS+Clt)/Δ*I* (PS) is given. The *x*‐fold current increase was determined as the value of the current amplitude after application of the stimulus (PS or PS+Clt) minus the value of the current amplitude under basal current conditions during whole‐cell patch clamp experiments. Measurements were performed in non‐transfected (NT), HEK‐TRPM3 (WT) and mutant‐transfected HEK293 cells. Significance levels were tested with a Mann–Whitney test and *P* values were corrected with the Bonferroni correction *P*/*n* with *n* = number of mutants; ^*^<2.9 × 10^−3^, ^*^^*^<5.9 × 10^−4^, ^*^^*^^*^< 5.9 × 10^−5^. NA: responses were not further analysed due to a lack of channel activity. †For the W982R mutant 100 μm PS was applied.

**Table 2:** Comparison of PS‐ and CIM0216‐induced activation of native TRPM3 and mutants

Channel	Segment	Δ*I*_−145mV_ PS (pA)	Δ*I*_−145mV_ CIM (pA)	Ratio Δ*I* (CIM)/Δ*I* (PS)	Significance
NT	–	NA	NA	–	–
WT	–	−54.3 ± 11.7	−8747.6 ± 1074.5	292.1 ± 62.3	–
W982R	S4	−3.0 ± 4.3	3.5 ± 0.9	−0.07 ± 0.2	**
W982F	S4	−87.6 ± 36.6	No response	0.01 ± 0.05	*
R985A	S4	−3.3 ± 0.9	No response	0.06 ± 1.3	*
R985Q	S4	−15.0 ± 5.6	No response	0.14 ± 0.14	**
D988R	S4	−43.8 ± 14.6	−18.7 ± 13.2	0.5 ± 0.3	0.003
D988K	S4	−3.3 ± 1.0	No response	−2.2 ± 4.7	**
D988E	S4	−38.6 ± 14.2	−8714.2 ± 2438.5	340.8 ± 142.8	0.93
G991R	S4	−373.3 ± 85.9	−5315.7 ± 1199.9	22.2 ± 6.0	**
G991A	S4	−127.8 ± 50.8	−6600.3 ± 1159.8	123.3 ± 31.1	0.038
Y981L	S4	−133.0 ± 31.0	−59.2 ± 20.9	0.5 ± 0.2	**
Y983V	S4	NA	NA	–	–
N993F	S4	−19.6 ± 10.0	−3795.8 ± 495.2	393.3 ± 107.6	0.38
Y878T	S1	−99.7 ± 55.9	No response	0.4 ± 0.79	*
Y882T	S1	−3752.6 ± 493.1	No response	−4.9E−4 ± 0.79	*
Y885T	S1	−87.2 ± 23.9	No response	−0.06 ± 0.02	0.003
F889L	S1	−12.4 ± 8.6	−1709.4 ± 289.9	402.9 ± 111.4	0.38
E941Q	S3	−62.7 ± 20.9	−5495.4 ± 757.6	119.3 ± 31.6	0.12
D947N	S3	NA	NA	–	–
D964N	S3	−272.6 ± 51.0	−8797.4 ± 1636.5	39.0 ± 8.3	*
D947R R985D	S3	NA	NA	–	–

The delta current increase at −145 mV (Δ*I*_−145mV_) induced by PS (40 μm) Δ*I* (PS) or by CIM0216 (1 μm) Δ*I* (CIM) and the ratio of the *x*‐fold current increases calculated as Δ*I* (CIM)/Δ*I* (PS) is given. The *x*‐fold current increase was determined as the value of the current amplitude after application of the stimulus (PS or CIM0216) minus the value of the current amplitude under basal current conditions during whole‐cell patch clamp experiments. Significance levels were tested with a Mann–Whitney test and *P* values were corrected with the Bonferroni correction *P*/*n* with *n* = number of mutants; ^*^<2.9 × 10^−3^, ^*^^*^<5.9 × 10^−4^, ^*^^*^^*^<5.9 × 10^−5^. NA: responses were not further analysed due to a lack of channel activity. No response indicates that CIM0216 showed no action on the mutant channel.

Together, these results demonstrate that mutation W982R in the VSD of TRPM3, which is at a position homologous to R1, abolishes currents through the non‐canonical pore, but not the canonical pore.

### Mutation of D988 (homologous to R3) to Arg or Lys prevents opening of the non‐canonical pore of TRPM3

**Figure 3:**
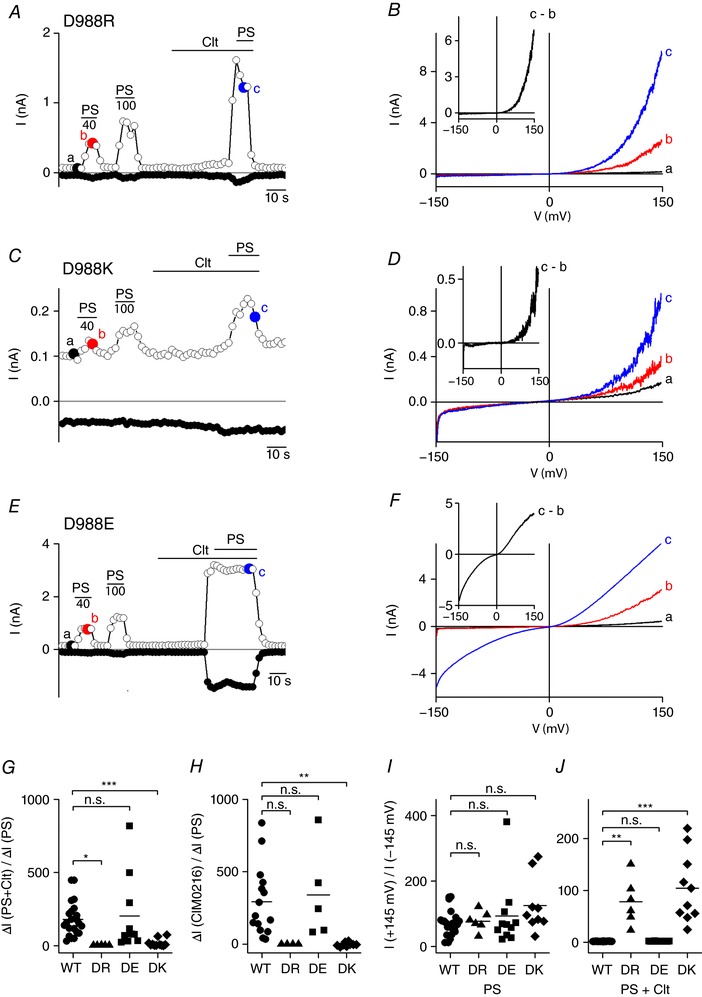
An arginine at position R3 abolishes the Clt+PS‐induced inward currents *A*, time course of whole‐cell currents at ±80 mV in HEK293 cells transiently expressing mutant D988R upon application of PS (40, 100 μm), Clt (10 μm) or PS+Clt (with PS 40 μm). *B*, *I–V* relationship of time points indicated in *A*. Inset, Clt‐induced current increase obtained as the difference between two traces in *B*. *C*, as in *A*, but in HEK293 cells transiently expressing mutant D988K. *D*, *I–V* relationship of time points indicated in *C*. *E* as in *A* and *C*, but in HEK293 cells transiently expressing mutant D988E. *F*, *I–V* relationship of time points indicated in *E*. *G*, *x*‐fold increase of the current response at −145 mV upon co‐application of Clt+PS normalized to the current response after stimulation by PS alone in TRPM3 (WT) and the mutants D988R (DR), D988E (DE) and D988K (DK). *H*, similar to *G*, but for CIM0216 application normalized to PS. *I*, PS‐induced rectification defined by the current at +145 mV divided by the current at −145 mV in TRPM3 (WT) and indicated mutants. *J*, Clt+PS‐induced rectification defined by the current at +145 mV divided by the current at −145 mV in TRPM3 and indicated mutants; Mann–Whitney test was used for statistical calculations and *P* values were corrected with the Bonferroni correction *P*/*n* with *n* = number of mutants; ^*^<2.9 × 10^−3^, ^**^<5.9 × 10^−4^, ^***^<5.9 × 10^−5^; n.s., not significant.

In the next step, an aspartate at position D988, a position equivalent to R3 of the Shaker K^+^ channel, was mutated to an arginine (D988R) (Fig. 1 *A* and *B*). The resulting D988R mutant exhibited normal activation by PS (40 μm) with a strongly outwardly rectifying *I–V* relationship, similar to wild‐type TRPM3 (Fig. 3 *A*, *B* and *I*, and Table 1). Remarkably, the inward current upon co‐application of Clt and PS was virtually abolished in the D988R mutant, resulting in a strongly outwardly rectifying *I–V* relationship (Fig. 3 *A*, *B*, *G* and *J*, and Table 1). Very similar results were obtained upon insertion of the positively charged lysine at this position (D988K), showing a complete lack of inward current activation in the presence of Clt (Fig. 3 *C*, *D*, *G* and *J*, and Table 1). In contrast, conservation of the negative charge at this position, by replacing the aspartate by a glutamate (D988E), yielded wild‐type‐like currents in both the absence and the presence of Clt (Fig. 3 *E*–*G*, *I* and *J*, and Table 1). Remarkably, application of CIM0216 to D988R and D988K did not induce any current increase at positive or negative membrane potentials. However, application of CIM0216 on D988E resulted in wild‐type‐like activation with strong current increases at positive and negative potentials, similar to the results in the presence of Clt+PS (Fig. 3 *H* and Table 2).

**Figure 4:**
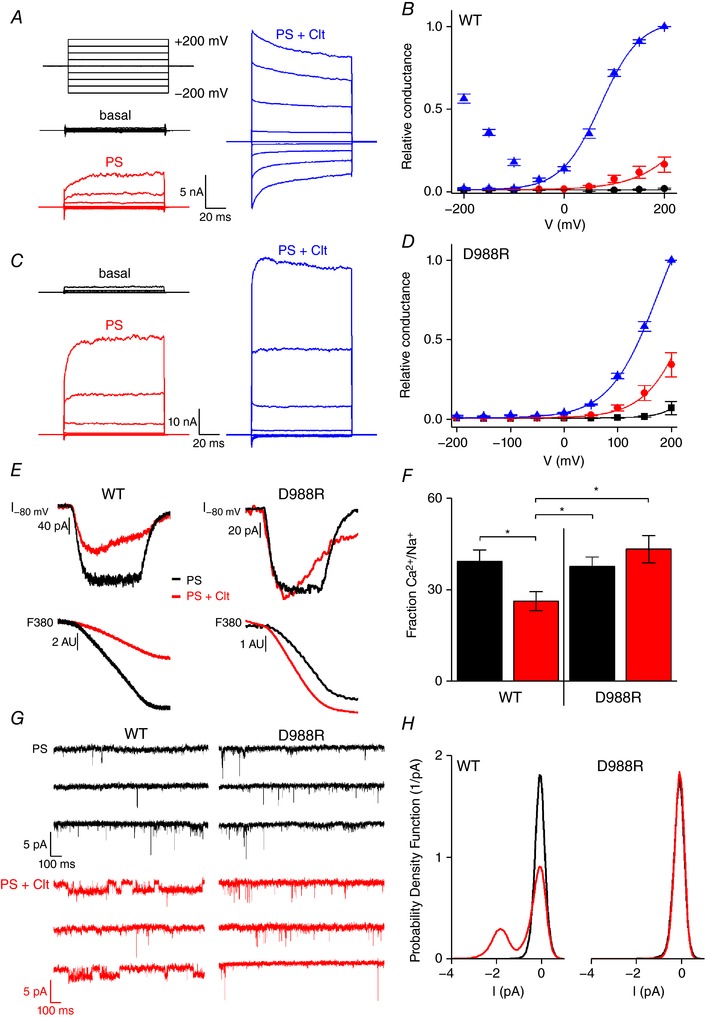
The D988R mutant showed no properties of the non‐canonical pore *A*, current traces of a single cell after applying a voltage‐step protocol ranging from −200 to +200 mV in +50 mV steps of 100 ms during basal conditions (black), application of 40 μm PS (red) and co‐application of PS (40 μm) and Clt (10 μm) (blue) in HEK‐TRPM3 cells. *B*, normalized *G–V* curves of TRPM3 (WT) with conductances normalized to the maximal conductance at +200 mV in the presence of PS+Clt (*n* = 5). Solid lines represent a global fit of a Boltzmann function (see Methods) to the data points at voltages ≥−50 mV. *C*, as in *A*, but in HEK293 cells transiently transfected with D988R. *D*, similar protocol to *B*, for the D988R mutant. *E*, fractional Ca^2+^ current measurements. Upper left panel shows measurements of ionic currents at −80 mV in HEK‐TRPM3 cells. Lower left panel illustrates simultaneous Fura_380_ measurements, exhibiting a decrease in signal that is linearly correlated to the amount of calcium influx. Cells were stimulated by PS (200 μm) (black traces) or by PS (40 μm) and Clt (10 μm) (red traces). Upper and lower right panel similar to left panels but in HEK293 cells overexpressing D988R mutant. *F*, fractional Ca^2+^ current of PS (black) and Clt+PS (red) induced TRPM3 currents in TRPM3 (WT) and D988R mutant (*n* ≥ 4 experiments per condition); Student's paired, two‐tailed *t* test was used for statistical comparison between data sets; ^*^<0.05. *G*, traces of single‐channel recordings in HEK293 cells transiently transfected with wild‐type TRPM3 or with the D988R mutant during application of PS (40 μm) (black traces) or during co‐application of PS (40 μm) + Clt (10 μm) (red traces). Shown traces were low‐pass filtered at 2 kHz. *H*, probability density function histograms of an example cell for wild‐type TRPM3 and the D988R mutant in presence of PS (black) or PS+Clt (red). To visualize the small conductance openings that are characteristic for the alternative ion permeation pathway in TRPM3, the high frequency signals that represent central pore openings of TRPM3 (Vriens *et al*. 2014) were abolished by low‐pass filtering prior to the histogram analyses.

Several points of evidence argue for the absence of the alternative ion permeation pathway in the single point mutant D988R upon co‐application of Clt+PS. First, activation of TRPM3 by PS (40 μm) results in a monophasic *G–V* curve due to the opening of the central pore (Fig. 4 *A* and *B*) (Vriens *et al*. 2014). Co‐application of Clt+PS results in a biphasic *G–V* curve in HEK‐TRPM3 cells, in line with the existence of two distinct pores with different conductivities: the central pore and the alternative non‐canonical pore (Vriens *et al*. 2014; Held *et al*. 2015). In contrast, stimulation of D988R with PS and by co‐application of Clt+PS results in currents with a single monophasic *G–V* curve (Fig. 4 *C* and *D*). Second, fractional Ca^2+^ measurements in HEK‐TRPM3 cells revealed a higher fractional Ca^2+^ current upon PS activation (39.30 ± 3.79%, *n* = 4) compared to co‐application of Clt+PS (26.30 ± 3.07%, *n* = 4) (Fig. 4 *E* and *F*). These results are in line with the presence of two distinct pores with different ion permeabilities as described earlier (Vriens *et al*. 2014). In contrast, no differences were observed in the fractional Ca^2+^ currents that permeate the D988R mutant upon PS stimulation (38 ± 3%, *n* = 4) and co‐application of Clt+PS (43 ± 4%, *n* = 6) (Fig. 3 *E* and *F*). Moreover, the fraction of the total inward current mediated by Ca^2+^ after co‐application of Clt+PS was similar to the wild‐type TRPM3 activated by PS alone (Fig. 4 *F*). Thirdly, Clt‐induced single‐channel openings were absent in the D988R mutant. Application of PS on wild‐type TRPM3 and D988R mutant showed typical short single‐channel openings of around 7 pA (Fig. 4 *G* upper panels), as was reported earlier (Vriens *et al*. 2014). Fittingly, co‐application of PS+Clt on native TRPM3 showed the appearance of additional single‐channel openings of around 2 pA (Fig. 4 *G* lower left panel), which were reported earlier as a characteristic of the non‐canonical ion pore (Vriens *et al*. 2014). However, co‐application of PS+Clt to D988R mutant did not induce the appearance of the 2 pA single‐channel currents (Fig. 4 *G* lower right panel). These results are further corroborated by the distinct probability density functions of wild‐type TRPM3 and D988R mutant channels (Fig. 4 *H*).

Altogether, these results indicate that insertion of an additional positive charge in the VSD of TRPM3 at position D988 prevents ion flux through the non‐canonical pore, whereas currents through the canonical pore are preserved.

### Mutation of G991 (homologous to R4) to larger amino acids reduces ionic currents through the non‐canonical pore of TRPM3

**Figure 5:**
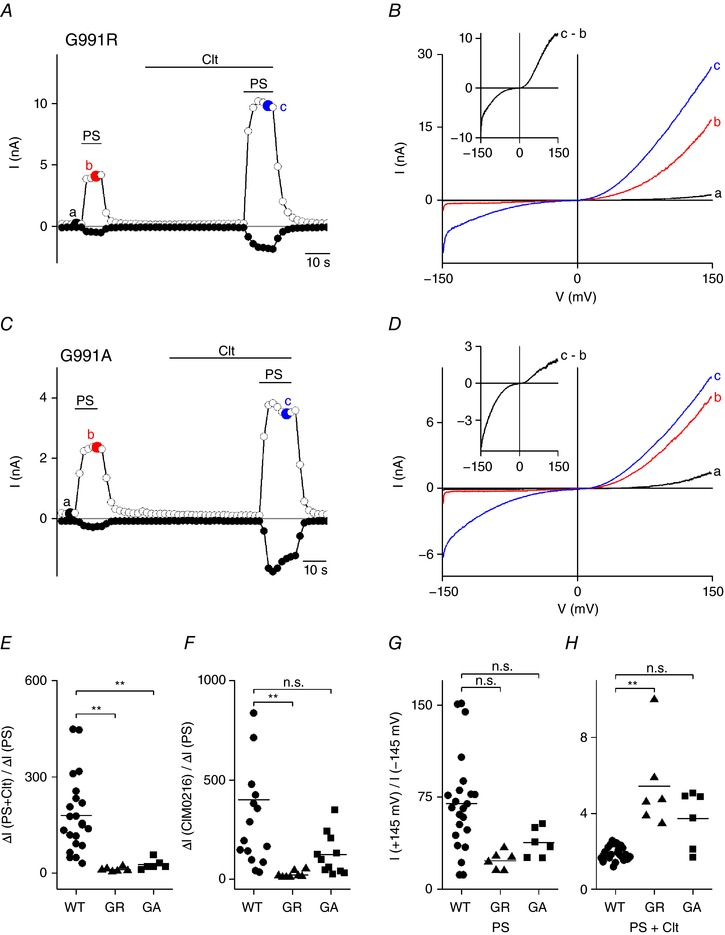
An arginine at position R4 hinders Clt+PS‐induced inward currents *A*, time course of whole‐cell currents at ±80 mV in HEK293 cells transiently expressing mutant G991R upon application of PS (40 μm), Clt (10 μm) or PS+Clt (with PS 40 μm). *B*, *I–V* relationship of time points indicated in *A*. Inset, Clt‐induced current increase obtained as the difference between two traces in *B*. *C*, similar protocol to that in *A* in HEK293 cells transiently expressing mutant G991A. *D*, *I–V* relationship of time points indicated in *C*. *E*, *x*‐fold increase of the current response at −145 mV upon co‐application of PS+Clt normalized to the current response upon single application of PS in TRPM3 (WT) and the mutants G991R (GR) and G991A (GA). *F*, similar to in *E*, but for CIM0216 application normalized to PS. *G*, PS‐induced rectification defined by the current at +145 mV divided by the current at −145 mV in the TRPM3 (WT) and indicated mutants. *H*, Clt+PS‐induced rectification defined by the current at +145 mV divided by the current at −145 mV in TRPM3 and indicated mutants; Mann–Whitney test was used for statistical calculations and *P* values were corrected with the Bonferroni correction *P*/*n* with *n* = number of mutants; ^*^<2.9 × 10^−3^, ^**^<5.9 × 10^−4^, ^***^<5.9 × 10^−5^; n.s., not significant.

**Figure 6:**
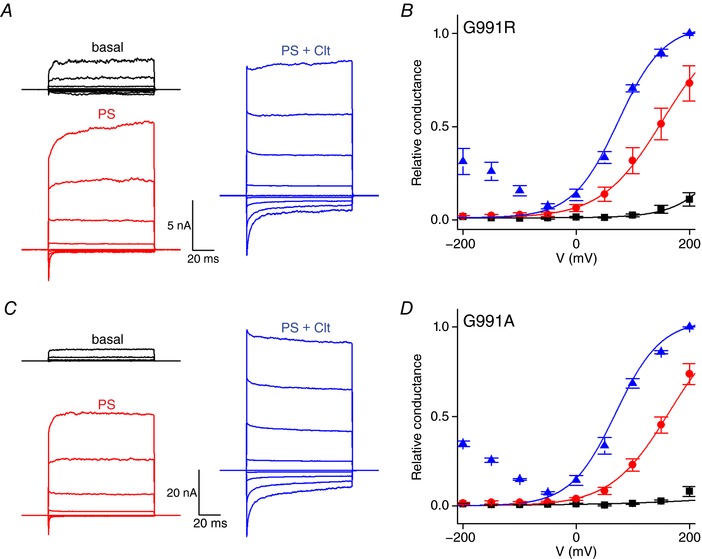
Replacing glycine‐991 with bulkier amino acids restricts the ionic currents through the non‐canonical pore *A*, current traces of a single cell after applying a voltage step protocol ranging from −200 to +200 mV in +50 mV steps of 100 ms during basal conditions (black), and application of 40 μm PS (red) and co‐application of Clt (10 μm) + PS (40 μm) (blue) in HEK293 cells transiently expressing the G991R mutant. *B*, normalized *G–V* curves of mutant G991R with conductances normalized to the maximal conductance at +200 mV in the presence of Clt+PS (*n* = 5). Solid lines represent a global fit of a Boltzmann function (see Methods) to the data points at voltages ≥−50 mV. *C*, similar to *A* in HEK293 cells transiently expressing G991A. *D* as in *B*, for the G991A mutant.

Subsequently, the glycine at the position corresponding to the Shaker R4 (G991) was replaced by an arginine (Fig. 1 *A* and *B*). Patch clamp experiments showed an outwardly rectifying *I–V* in G991R‐transfected HEK293 cells upon PS stimulation (40 μm) (Fig. 5 *A*, *B* and *G*). Upon co‐application of Clt and PS or stimulation by CIM0216, a robust inward current was evoked (Fig. 5 *A* and *B*, and Tables 1 and 2). However, the rectification scores were increased (5.42 ± 0.97 for Clt+PS) and the relative increase in inward current significantly decreased compared to WT TRPM3 (22.2 ± 6.0 for Clt+PS) (Fig. 5 *E*, *F* and *H*, and Tables 1 and 2). Substituting an alanine at this position (G991A) caused a similar but less pronounced effect, with a significant reduction of the potentiation of the PS‐induced currents by Clt (rectification_+145/−145_: 3.73 ± 0.61; ratio_Δ_ *_I_*_(PS+Clt)/Δ_ *_I_*_(PS)_: 26.8 ± 6.6) (Fig. 5 *C*–*E* and *H*, and Table 1) and a reduction of the current increase ratio at −145 mV of CIM0216 and PS (WT: 292.1 ± 62.3; G991A: 123.3 ± 31.1) (Fig. 5 *F* and Table 2). Next, we investigated whether mutations of G991 affected the typical biphasic *G–V* curve of the native TRPM3 channel after co‐application of Clt+PS. Stimulation of both G991R and G991A mutant channels by PS resulted in a monophasic *G–V* curve, while co‐application of Clt+PS resulted in a biphasic *G–V* curve (Fig. 6 *A*–*D*). However, the relative conductance at hyperpolarizing membrane potentials (−200 mV) was significantly reduced in both mutant channels G991R and G991A compared to wild‐type TRPM3 (WT: 0.56 ± 0.03; G991R: 0.31 ± 0.07; G991A: 0.35 ± 0.02). These results suggest that inward currents through the non‐canonical pore are significantly reduced by insertion of larger amino acid residues at position G991.

### The conserved R2 arginine at position 985 is crucial for the occurrence of non‐canonical pore currents

**Figure 7:**
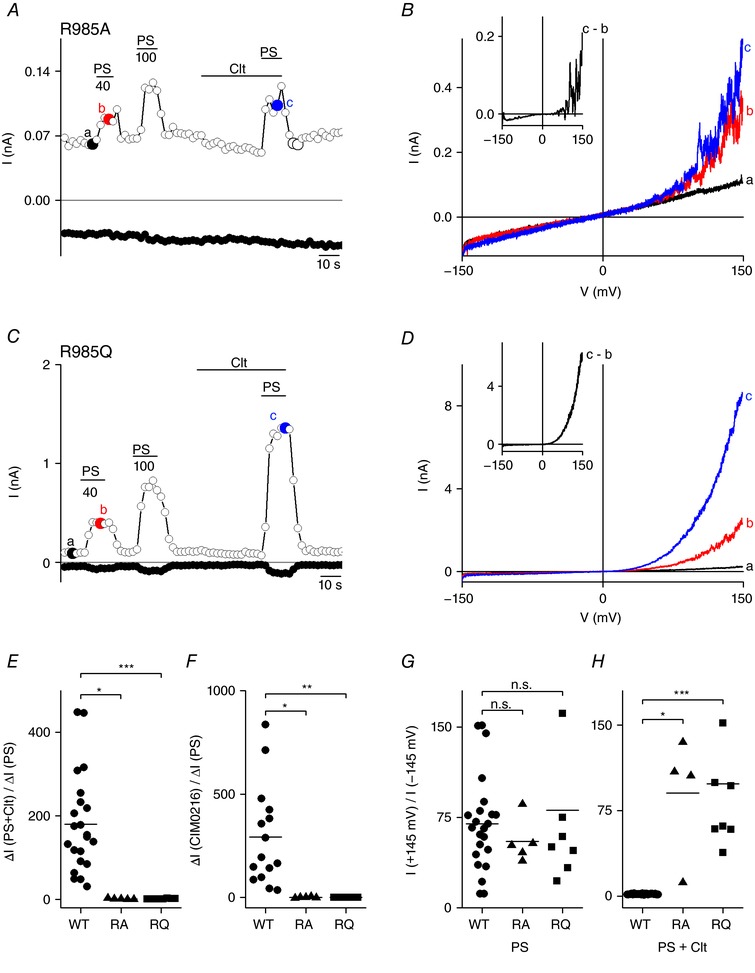
The arginine at position R2 is crucial for the occurrence of the Clt+PS‐induced inward currents *A*, time course of whole‐cell currents at ±80 mV in HEK293 cells transiently expressing mutant R985A upon application of PS (40, 100 μm), Clt (10 μm) or PS+Clt (with PS 40 μm). *B*, *I–V* relationship of time points indicated in *A*. Inset, Clt‐induced current increase obtained as the difference between two traces in *B*. *C*, similar protocol to that in *A* in HEK293 cells transiently expressing mutant R985Q. *D*, *I–V* relationship of time points indicated in *C*. *E*, *x*‐fold increase of the current response at −145 mV upon co‐application of PS+Clt normalized to the current response upon single application of PS in TRPM3 (WT) and the mutants R985A (RA) and R985Q (RQ). *F*, similar to *E*, but for CIM0216 application normalized to PS. *G*, PS‐induced rectification defined by the current at +145 mV divided by the current at −145 mV in the TRPM3 (WT) and indicated mutants. *H*, Clt+PS‐induced rectification defined by the current at +145 mV divided by the current at −145 mV in TRPM3 and indicated mutants; Mann–Whitney test was used for statistical calculations and *P* values were corrected with the Bonferroni correction *P*/*n* with *n* = number of mutants; ^*^<2.9 × 10^−3^, ^**^<5.9 × 10^−4^, ^***^<5.9 × 10^−5^; n.s., not significant.

Neutralizing R985 residue by replacement with alanine (R985A) resulted in a mutant channel that was poorly responsive to stimulation with PS (40–100 μm) or co‐application of Clt and PS (Fig. 7 *A* and *B*, and Table 1). Moreover, this mutant was non‐responsive to stimulation by CIM0216 (Fig. 7 *F* and Table 2). Likewise, substitutions of lysine (R985K) or histidine (R985H) resulted in mutant channels that showed very modest PS‐induced increases in current amplitudes, which were difficult to differentiate from background currents (Table 1). Therefore, these data indicate that Arg at position 985 plays a crucial role in the normal gating of the channel. Nevertheless, patch clamp data indicated the abolition of the Clt‐induced inward currents in R985A, resulting in an increased rectification score (90.3 ± 26.94) and a decreased *x*‐fold ratio of the inward currents (ratio_Δ_ *_I_*_(PS+Clt)/Δ_ *_I_*_(PS)_: 1.2 ± 0.6) (Fig. 7 *A*, *B*, *E* and *H*, and Table 1). Notably, insertion of a glutamine at position 985 (R985Q) resulted in a mutant channel that produced robust PS‐induced currents but lacked the Clt‐induced inward currents. Indeed, patch clamp experiments showed an outwardly rectifying *I–V* in R985Q‐transfected HEK293 cells upon PS stimulation (rectification_+145/−145_: 80.8 ± 22.54; Fig. 7 *C*, *D* and *G*). Co‐application of PS+Clt resulted in an outwardly rectifying *I–V* lacking the Clt‐induced inward currents (rectification_+145/−145_: 98.4 ± 21.62; ratio_Δ_ *_I_*_(PS+Clt)/Δ_ *_I_*_(PS)_: 1.5 ± 0.3; Fig. 7 *C*–*E* and *H*, and Table 1). In fact, in comparison to WT the rectification score was significantly increased in the presence of PS+Clt (Fig. 7 *H* and Table 1), while CIM0216 was without effect on the R985Q mutant (Fig. 7 *F* and Table 2).

To conclude, mutations at position 985 resulted in a reduced channel activity and all designed mutants at that position led to the abolition of the Clt‐induced inward currents upon co‐application of PS+Clt. These results suggest that arginine 985 is a key residue for the occurrence of the non‐canonical pore currents.

### Mutation of residues located at non‐gating charge positions in S4 influence the non‐canonical pore currents induced by Clt+PS

Next, the involvement of residues in S4 of TRPM3 at positions that correspond to non‐gating charge residues in Shaker was evaluated. Therefore, residues in S4 with no conservation to their aligned residue in the Shaker K^+^ channel were mutated to the equivalent amino acid in the Shaker channel (Y981L at R1 −1, Y983V at R1 +1 and N993F at R4 +2; Fig. 1 *A*). Mutation of Y981 to a leucine (Y981L) resulted in a channel that was responsive to PS stimulation. Surprisingly, activation by PS alone induced a prominent inward current that was never observed in wild‐type TRPM3, resulting in a rectification score of 4.8 ± 1.04 (Table 1). However, the PS‐induced inward current was sensitive to the central pore blocker La^3+^ (10 μm), resulting in a 100% block at −80 mV (data not shown). Remarkably, co‐application of PS+Clt did not induce any potentiation of the PS‐induced inward and outward currents (ratio_Δ_ *_I_*_(PS+Clt)/Δ_ *_I_*_(PS)_: 0.4 ± 0.1) (Table 1). Note however, that currents in Y981L were subject to a fast time‐dependent desensitization, which might have hidden a Clt‐induced current potentiation. In addition, stimulation of Y981L by CIM0216 resulted in an outwardly rectifying *I–V* relationship without the presence of a prominent inward current (ratio_Δ_ *_I_*_(CIM)/Δ_ *_I_*_(PS)_: 0.5 ± 0.2; Table 2). Next, Y983 was mutated to a valine (Y983V), which resulted in a mutant channel that was not responsive to PS, PS+Clt or CIM0216 stimulation. Finally, N993 was replaced by a phenylalanine (N993F), resulting in a channel that was responsive to PS with a normal *I–V* curve (rectification_+145/−145_: 64.0 ± 24.77; Table 1). Further, co‐application of Clt+PS resulted in robust inward and outward currents. However, the Clt‐induced current potentiation was significantly decreased (ratio_Δ_ *_I_*_(PS+Clt)/Δ_ *_I_*_(PS)_: 20.9 ± 7.1; Table 1) and the rectification score was increased compared to wild‐type (rectification_+145/−145_: 13.0 ± 2.01). Surprisingly, application of CIM0216 induced robust inward and outward currents in the N993F mutant, resulting in a potentiation ratio that was not significantly different from the WT (Table 2).

Altogether, these results propose a minor role of non‐gating charge residues in S4 in the determination of the properties and opening of the non‐canonical pore in TRPM3.

### Residues in transmembrane segment S1 play a crucial role in the occurrence of non‐canonical pore currents

**Figure 8:**
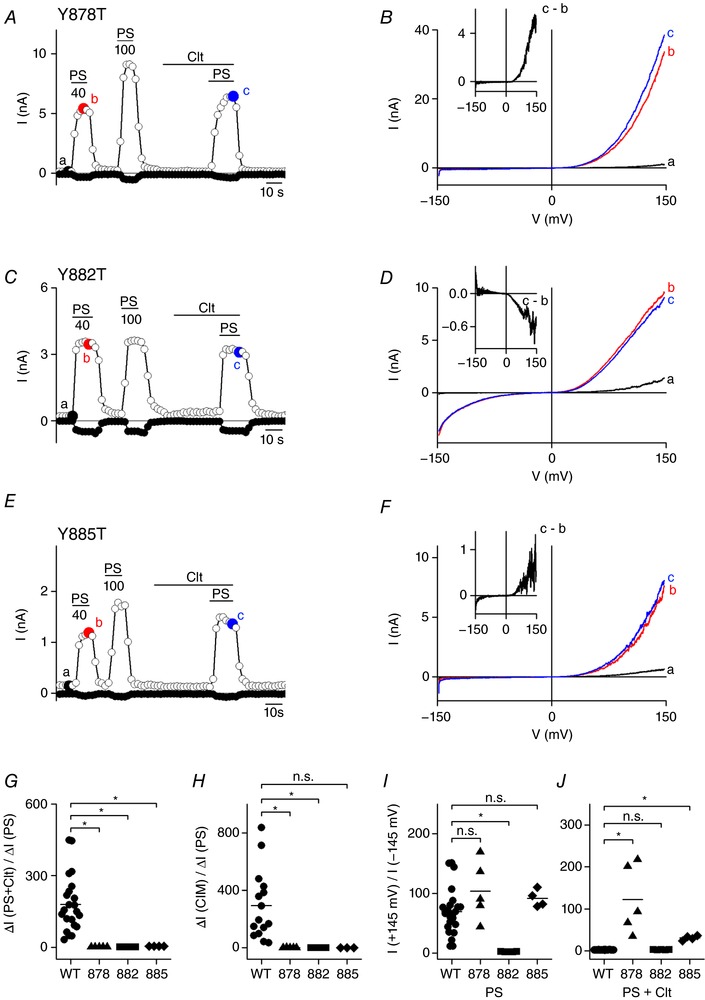
Three aromatic residues in S1 are crucial for the occurrence of the Clt+PS‐induced inward currents *A*, time course of whole‐cell currents at ±80 mV in HEK293 cells transiently expressing mutant Y878T upon application of PS (40, 100 μm), Clt (10 μm) or PS+Clt (with PS 40 μm). *B*, *I–V* relationship of time points indicated in *A*. Inset, Clt‐induced current increase obtained as the difference between two traces in *C*. *C*, similar protocol to that in *A* in HEK293 cells transiently expressing mutant Y882T. *D*, *I–V* relationship of time points indicated in *C*. *E*, similar protocol to that in *A* and *C* in HEK293 cells transiently expressing mutant Y885T. *F*, *I–V* relationship of time points indicated in *E*. *G*, *x*‐fold increase of the current response at −145 mV upon co‐application of PS+Clt normalized to the current response upon single application of PS in TRPM3 (WT) and the mutants Y878T (878), Y882T (882) and Y885T (885). *H*, similar to *G*, but for CIM0216 application normalized to PS. *I*, PS‐induced rectification defined by the current at +145 mV divided by the current at −145 mV in TRPM3 (WT) and indicated mutants. *J*, Clt+PS‐induced rectification defined by the current at +145 mV divided by the current at −145 mV in TRPM3 and indicated mutants; Mann–Whitney test was used for statistical calculations and *P* values were corrected with the Bonferroni correction *P*/*n* with *n* = number of mutants; ^*^<2.9 × 10^−3^, ^**^<5.9 × 10^−4^, ^***^<5.9 × 10^−5^; n.s., not significant.

Finally, the impact of other transmembrane segments on the occurrence of the non‐canonical pore currents was investigated. To determine additional residues in the VSD involved in the gating of the alternative ion permeation pathway of TRPM3, a homology model was designed, based on the cryo‐EM structure of TRPM4 (Guo *et al*. 2017). The homology model indicated four aromatic residues in S1 orientated to the inner core of the VSD, namely Y878, Y882, Y885 and F889 (Fig. 1 *B*). The position of these residues in the homology model proposed a potential interaction with the W982 (R1) in S4. Subsequently, single point‐mutations were designed in which all four aromatic residues were changed to the non‐aromatic threonine. Interestingly, the Clt‐induced activation of inward currents was abolished in the Y878T, Y882T and Y885T mutant channels, resulting in a significantly decreased ratio of the current increase (Fig. 8 *A*–*G* and Table 1). Moreover, these mutants did not show any current increase after stimulation by CIM0216 (Fig. 8 *H* and Table 2). Interestingly, in the Y882T mutant prominent inward currents upon sole application of PS were observed (Fig. 8 *C*, *D* and *I*, and Table 1). These currents did not further increase in the presence of Clt+PS and were sensitive to the block by the non‐specific pore blocker La^3+^ (102.1 ± 8.54% block at −80 mV; data not shown). Mutation of the fourth aromatic residue, F889, to leucine resulted in a PS‐, PS+Clt‐ and CIM0216‐induced response that was similar to wild‐type TRPM3 (Tables 1 and 2).

Additionally, three residues in the putative S3 region of TRPM3 were mutated. It has been reported that negatively charged residues in S1–S3 of the Shaker potassium channel form salt bridges with the gating charge arginines in S4, which stabilizes the S4 segment and thereby facilitates voltage sensor movement (Papazian *et al*. 1995; Tao *et al*. 2010). Interestingly, our homology model proposed a proximity of D947 in S3 with R985 in S4 (Fig. 1 *B*). Consistent with the possible importance of this charge interaction, the D947R mutation showed no channel activity after stimulation by PS and PS+Clt (Table 1). Note that mutation of R985 to other amino acids often resulted in strongly disabled channels with low current amplitudes (Table 1). To further investigate the possible formation of a salt bridge between D947 and R985, a charge swap mutation D947R/R985D was designed. However, this mutant showed only minimal channel activity upon stimulation by PS or PS+Clt. Therefore, the presence of a salt bridge between R985 and D947 could not be confirmed in our experiments. Subsequently, other negatively charged residues in the S3 segment (E941 and D964), which are more distant from R985 in our homology model, were neutralized by replacement with uncharged residues (E941Q and D964N). Interestingly, both mutants showed PS sensitivity in HEK293 expressing cells with strong inward currents at negative membrane potentials when treated with PS+Clt or CIM0216, consistent with a less important role of these residues in the possible charge interaction with R985. However, statistical analysis revealed a significant reduction of these inward currents in comparison to WT TRPM3 (ratio_Δ_ *_I_*_(PS+Clt)/Δ_ *_I_*_(PS)_ in E941Q: 34.7 ± 7.5; in D964N: 24.4 ± 2.4) (Table 1).

To conclude, additional residues in the VSD outside the S4 of TRPM3 were identified as important residues involved in the appearance and gating of the non‐canonical pore.

## Discussion

In earlier work, evidence was provided for the presence of a non‐canonical ion permeation pathway in TRPM3, distinct from the central pore, which can be opened by co‐application of PS+Clt or by stimulation with CIM0216 (Vriens *et al*. 2014; Held *et al*. 2015). Currently, knowledge on the localization of the non‐canonical pore within the TRPM3 channel protein is very scarce. By the use of mutagenesis studies and homology modelling, we have identified critical residues within the VSD that strongly affect ionic currents along the alternative ion permeation pathway in TRPM3.

Alignment of the S4 segments of TRPM3 and Shaker K^+^ channel revealed that only a single arginine at the R2 position is conserved, equivalent to position 985 in TRPM3. The presence of only a single positively charged residue in the S4 may explain the existence of a naturally occurring non‐canonical current in the native TRPM3 channel. In support of that notion, further mutagenesis studies showed that insertion of positively charged residues at positions corresponding to R1, R3 or R4 had a strong inhibitory effect on the inward currents in the presence of Clt and PS.

Indeed, introduction of an arginine at the position corresponding to R1 of the Shaker K^+^ channel (W982R) disrupts the Clt+PS‐induced inward current as was reported in previous studies (Vriens *et al*. 2014; Held *et al*. 2015). In contrast, a more conservative substitution of the tryptophan to phenylalanine resulted in a mutant channel (W982F) with similar *I*–*V* properties as the WT after stimulation by PS and Clt+PS. In line with these results, insertion of a positively charged arginine or lysine at the position corresponding to R3 in the Shaker K^+^ channel (D988R and D988K) resulted in similar absence of PS+Clt‐induced inward currents, while conservative change of the aspartate to a glutamate mimicked WT‐like currents upon co‐stimulation with PS+Clt. Furthermore, introduction of an arginine or an alanine at position R4 (G991R and G991A) resulted in significantly reduced inward currents after co‐application of Clt+PS. The reduction of the inward currents in these two mutants could be explained by the enlarged size of the incorporated amino acid from glycine < alanine < arginine, which might reduce the inward currents either through sterical hindrance or through a reduction of the flexibility of the C‐terminal end of S4.

Altogether, these results indicate a critical role of the amino acids at position R1, R3 and R4 in TRPM3 for the existence of a non‐canonical pore. In fact, these results are in line with published results on voltage‐gated K^+^, Na^+^ and Ca^2+^ channels, in which the non‐canonical pores could only be formed by the connection of the outer and inner water crevices in the voltage‐sensor upon replacement of the gating charges by smaller, uncharged amino acids (Starace *et al*. 1997; Starace & Bezanilla, 2004; Tombola *et al*. 2005; Held *et al*. 2016). These results are also in line with studies of the proton channel H_V_1 that possesses a natural non‐canonical pore that can be blocked by introduction of an arginine at position R4 in the S4 voltage sensor (Tombola *et al*. 2008). Moreover, certain disease‐causing mutations in known channelopathies are linked to mutations at position R1, R3 and R4 of voltage‐gated channels. For example, hypokalaemic periodic paralysis is caused by R1 mutations in Na_v_1.4 (R669H; Struyk & Cannon, 2007) and Ca_v_1.1 (R528H; Wu *et al*. 2012) channels. In addition, mixed arrhythmias and dilated cardiomyopathy can be explained by the presence of a non‐canonical pore caused by a point mutation in the Na_v_1.5 channel at position R1 (R219H; Gosselin‐Badaroudine *et al*. 2012). Further, mutations of the arginines at position R3 in Na_v_1.4, Na_v_1.5 and Ca_v_1.1 were also associated with non‐canonical pore currents and the above‐mentioned diseases (for review see Held *et al*. 2016). Finally, arginine mutations at position R4 of hK_V_7.2 were connected to neuronal hyperexcitability (Miceli *et al*. 2012).

Interestingly, mutating the only conserved arginine at position R2 in S4 of TRPM3 to other amino acids resulted in channels that also did not show Clt‐induced inward currents upon co‐application of PS+Clt. Although the abolition of non‐canonical pore currents upon neutralization of an arginine in S4 might seem surprising in respect to the mentioned studies in other voltage‐gated channels, it is well in line with a study reporting alternative ionic currents through the VSD of a truncated Shaker K^+^ channel, still possessing all S4 gating charges but lacking S5–S6 (Zhao & Blunck, 2016). Thus, the existence of positively charged residues in S4 can pose a hindrance for the occurrence of non‐canonical currents through the VSD in voltage‐gated channels, but is not an exclusion criterion and strongly depends on the structural features of the ion channel in question.

Additionally, this study identified other important regions outside S4 involved in the occurrence of the non‐canonical pore in TRPM3. First, we identified a cluster of aromatic residues in the S1 region based on a homology model of TRPM3. It encompasses a threesome of tyrosine residues with proposed proximity to the tryptophan at position 982 of S4. Indeed, neutralization of any of the three aromatic residues in S1 (Y878, Y882 and Y885) resulted in functional channels that responded to PS, but did not show Clt‐induced inward currents. In addition, all three mutants showed minor Clt‐induced outward currents and were insensitive to CIM0216 stimulation. These results suggest that the tyrosines either form an important part of the ligand interaction site for Clt and CIM0216 or are involved in the gating of the non‐canonical pore following binding of Clt and CIM0216.

A second important region involved in the properties of the alternative ion permeation pathway was identified in transmembrane segment 3. Neutralization of negative residues in S3 (E941Q and D964N) resulted in reduced Clt‐ and CIM0216‐induced inward currents, suggesting either a mutation‐induced hindrance of smooth voltage‐sensor movement or an influence on the polarity of the water crevices within the non‐canonical pore (hindrance of cation flow) due to spatial proximity of the mutated residues to the pathway. Note that amino acids in S1–S3 have also been reported to play a crucial role in voltage‐sensor movement and ion permeability and selectivity in other ion channels, possessing non‐canonical pores, including the Shaker K^+^ channel and H_V_1 (Papazian *et al*. 1995; Tao *et al*. 2010; Berger & Isacoff, 2011; Musset *et al*. 2011; Pless *et al*. 2011; Lacroix *et al*. 2014).

Structural modelling can be a powerful tool to obtain a broader idea of the global organization of the protein of interest. However, a homology model cannot replace a valid channel structure obtained experimentally. Nevertheless, the homology model of TRPM3, based on the closely related TRPM4 cryo‐EM structure (PDB: 6bco; Guo *et al*. 2017), enabled us to identify critical residues in S1 and S3 involved in the gating of the non‐canonical pore of TRPM3 (Fig. 1 *B*).

Alternatively, some of the mutagenesis results can also be explained as a modification of the agonist interaction sites, thereby influencing the affinity of the ligand binding, or by an allosteric effect of the mutagenesis on the central pore. Although the PS‐interaction site is suggested to be located at the outside of the TRPM3 protein (Wagner *et al*. 2008; Vriens *et al*. 2014), we cannot rule out this possibility since it is known that other ligands can interact with the S4 segments of related TRP channels (Voets *et al*. 2007; Vriens *et al*. 2007). Furthermore, clotrimazole is suggested to permeate the plasma membrane in inside‐out patches, which may allow it to interact with the S4 segment of the VSD and thereby increase the opening time of the non‐canonical pore. However, several of our results argue against one of the above‐mentioned mechanisms and rather propose a direct influence of the mutations on the non‐canonical pore itself. Firstly, the concentration‐dependent effect of PS (tested at different doses of 40 and 100 μm) was not altered in most of the mutants compared to native TRPM3. Secondly, the rectification score of the PS‐induced currents is mainly stable even though the PS+Clt‐induced rectification was altered. Thirdly, the Clt‐induced potentiation of the outward currents was for most mutants still intact, even when the inward current potentiation was abolished, suggesting that Clt interaction was not crucially affected. Fourthly, the inward rectifying currents, typically a hallmark for the existence of the alternative pathway, were not abolished when the amino acids were mutated to an analogous charged residue. It should be noted, however, that stimulation by CIM0216 did not always result in an activation of the TRPM3 mutants (Table 2), arguing for a potential abolition of CIM0216 interaction or CIM0216‐induced gating of TRPM3. Earlier studies reported a competitive interaction between CIM0216 and Clt, and therefore an overlap of the binding sites can be speculated to occur (Held *et al*. 2015). However, for some mutants reduced or no Clt‐induced inward currents were observed, while CIM0216‐induced channel activation remained intact and resulted in similar reduction or abolition of the inward current potentiation compared to wild‐type TRPM3.

Additional experiments are required to investigate whether the designed mutations in the VSD affect the PS, Clt and CIM0216 affinity of TRPM3, the gating mechanism of the non‐canonical pore or disturb the ionic permeation through the non‐canonical pore due to steric or charge hindrance.

**Figure 9:**
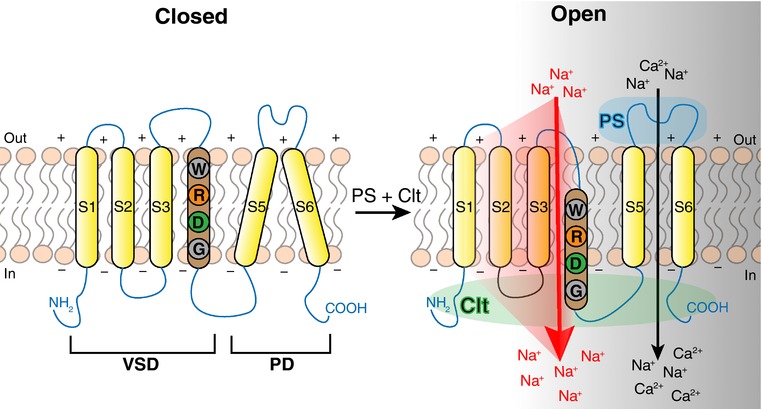
Model of the location of the alternative ion permeation pathway in TRPM3 The cartoon illustrates the proposed location and opening mechanism of the non‐canonical ion permeation pathway in TRPM3 during hyperpolarization. The transmembrane segment 4 (S4) is shown in brown. Corresponding positions of R1 to R4 are shown as follows: W: position W982, correlating to position R1; R: position R985, corresponding to position R2; D: position D988, corresponding to position R3; and G: position G991, correlating with position R4. Colour code indicating charges of amino acids: grey, neutral; orange, positive; green, negative. PS binding is indicated as blue cloud at the extracellular side of the membrane and Clt binding is indicated as green cloud at the intracellular side of the membrane. Black arrow indicates the canonical pore of TRPM3; red arrow and shadow indicate the non‐canonical ion permeation pathway in TRPM3 within the VSD in proximity to S4.

Based on the TRPM3 homology model and the obtained mutagenesis results we propose a mechanism for the opening of the non‐canonical pore as shown in Fig. 9. We suggest that the non‐canonical pore of TRPM3 is established by movement of S4 during membrane hyperpolarization. A similar mechanism has been proposed for the opening of the non‐canonical pore in voltage‐gated K^+^, Na^+^ and Ca^2+^ channels as well as for the pore opening of the VSD protein H_V_1 during membrane depolarization (for review see Held *et al*. 2016). The S4 segment of voltage‐gated K^+^, Na^+^ and Ca^2+^ channels contains a series of at least three positively charged residues, while the transmembrane segments S1, S2 and S3 contain organized structures of highly conserved negatively charged amino acids that appear to be involved in the stabilization of S4 in different conformational states (Pless *et al*. 2011). At the centre of each VSD is the gating charge transfer centre (GCTC) that separates the intra‐ and extracellular water crevices (Tao *et al*. 2010). Neutralizing the positively charged amino acids of S4 that interact with the gating charge transfer centre located at the centre of each VSD will change the hydrophobicity of the GCTC and thereby create an ion‐permeating non‐canonical pore (Starace & Bezanilla, 2004; Tombola *et al*. 2007; Gamal El‐Din *et al*. 2010). Equally, H_V_1 possesses an S4 segment with three Arg gating charge residues (R1–R3). The amino acid at position R4 is an asparagine (N214) that when mutated to an Arg abolishes the proton flux through H_V_1 (Tombola *et al*. 2008). Possibly, PS binding on TRPM3 may affect the motional freedom of S4. The PS‐induced modification of S4 movement together with a possible effect of clotrimazole on the hydrophobicity of a putative GCTC may allow S4 to move within the VSD during membrane hyperpolarization and as such open the non‐canonical pore in TRPM3 (Fig. 9). However, additional studies are required to provide further evidence for the proposed mechanism underlying the opening of the non‐canonical pore (e.g. voltage fluorometry measurements to determine the movements of the different segments in the VSD).

In conclusion, this study provides additional evidence for the existence of a non‐canonical pore in TRPM3, through the identification of a range of amino acid residues in the VSD that are required for its proper function and/or occurrence (Fig. 1 *B*). These results are in line with the notion that similar structures underlie the non‐canonical pore in TRPM3 and in voltage‐gated K^+^, Na^+^ and Ca^2+^ channels and the proton channel H_V_1. These results enhance our fundamental knowledge of non‐canonical pores, and could be valuable for the development of specific inhibitors of the alternative pore, to treat specific types of pain.

## Additional information

### Competing interests

The authors declare no competing interests.

### Author contributions

This work was performed in the Laboratory of Ion Channel Research at the KU Leuven. K.H., T.V. and J.V. designed the work, K.H., F.G., D.A. and A.J. acquired and analysed the data, K.H., T.V. and J.V. interpreted the data, K.H., F.G., C.U., T.V. and J.V. drafted the manuscript and F.G., C.U, T.V. and J.V. revised the manuscript critically. All authors have read and approved the final version of this manuscript and agree to be accountable for all aspects of the work in ensuring that questions related to the accuracy or integrity of any part of the work are appropriately investigated and resolved. All persons designated as authors qualify for authorship, and all those who qualify for authorship are listed.

### Funding

This project has received funding from the Belgian Federal Government (IUAP P7/13 to T.V.), the Research Foundation‐Flanders (G.0565.07, G.0825.11 and G084515N to T.V. and J.V.), the Research Council of the KU Leuven (IOF‐HB/12/023 to J.V. and T.V. and PF‐TRPLe to C.U. and T.V.), and the European Union's Horizon 2020 research and innovation programme under the Marie Sklodowska‐Curie grant agreement No 665501 with the research Foundation Flanders (FWO). F.G. is a ‘FWO [PEGASUS]^2^ Marie Sklodowska‐Curie Fellow’ and K.H. is a ‘Postdoctoral Fellow’ of the Research Foundation – Flanders, Belgium.

## References

[tjp12942-bib-0001] Aggarwal SK & MacKinnon R (1996). Contribution of the S4 segment to gating charge in the Shaker K^+^ channel. Neuron 16, 1169–1177. 8663993 10.1016/s0896-6273(00)80143-9

[tjp12942-bib-0002] Benkert P , Biasini M & Schwede T (2011). Toward the estimation of the absolute quality of individual protein structure models. Bioinformatics 27, 343–350. 21134891 10.1093/bioinformatics/btq662 PMC3031035

[tjp12942-bib-0003] Berger TK & Isacoff EY (2011). The pore of the voltage‐gated proton channel. Neuron 72, 991–1000. 22196334 10.1016/j.neuron.2011.11.014 PMC3244940

[tjp12942-bib-0004] Bezanilla F (2000). The voltage sensor in voltage‐dependent ion channels. Physiol Rev 80, 555–592. 10747201 10.1152/physrev.2000.80.2.555

[tjp12942-bib-0005] Bezanilla F (2002). Voltage sensor movements. J Gen Physiol 120, 465–473. 12356849 10.1085/jgp.20028660 PMC2229527

[tjp12942-bib-0006] Biasini M , Bienert S , Waterhouse A , Arnold K , Studer G , Schmidt T , Kiefer F , Gallo Cassarino T , Bertoni M , Bordoli L & Schwede T (2014). SWISS‐MODEL: modelling protein tertiary and quaternary structure using evolutionary information. Nucleic Acids Res 42, W252‐258. 24782522 10.1093/nar/gku340 PMC4086089

[tjp12942-bib-0007] Catterall WA (2010). Ion channel voltage sensors: structure, function, and pathophysiology. Neuron 67, 915–928. 20869590 10.1016/j.neuron.2010.08.021 PMC2950829

[tjp12942-bib-0008] Gamal El‐Din TM , Heldstab H , Lehmann C & Greeff NG (2010). Double gaps along Shaker S4 demonstrate omega currents at three different closed states. Channels (Austin) 4, 93–100. 20009570

[tjp12942-bib-0009] Gosselin‐Badaroudine P , Keller DI , Huang H , Pouliot V , Chatelier A , Osswald S , Brink M & Chahine M (2012). A proton leak current through the cardiac sodium channel is linked to mixed arrhythmia and the dilated cardiomyopathy phenotype. PLoS One 7, e38331. 22675453 10.1371/journal.pone.0038331 PMC3365008

[tjp12942-bib-0010] Grimm C , Kraft R , Sauerbruch S , Schultz G & Harteneck C (2003). Molecular and functional characterization of the melastatin‐related cation channel TRPM3. J Biol Chem 278, 21493–21501. 12672799 10.1074/jbc.M300945200

[tjp12942-bib-0011] Guo J , She J , Zeng W , Chen Q , Bai XC & Jiang Y (2017). Structures of the calcium‐activated, non‐selective cation channel TRPM4. Nature 552, 205–209. 29211714 10.1038/nature24997 PMC5901961

[tjp12942-bib-0012] Held K , Kichko T , De Clercq K , Klaassen H , Van Bree R , Vanherck JC , Marchand A , Reeh PW , Chaltin P , Voets T & Vriens J (2015). Activation of TRPM3 by a potent synthetic ligand reveals a role in peptide release. Proc Natl Acad Sci USA 112, E1363–E1372. 25733887 10.1073/pnas.1419845112 PMC4371942

[tjp12942-bib-0013] Held K , Voets T & Vriens J (2016). Signature and pathophysiology of non‐canonical pores in voltage‐dependent cation channels. Rev Physiol Biochem Pharmacol 170, 67–99. 26791748 10.1007/112_2015_5003

[tjp12942-bib-0014] Horn R (2002). Coupled movements in voltage‐gated ion channels. J Gen Physiol 120, 449–453. 12356847 10.1085/jgp.20028658 PMC2229539

[tjp12942-bib-0015] Lacroix JJ , Hyde HC , Campos FV & Bezanilla F (2014). Moving gating charges through the gating pore in a Kv channel voltage sensor. Proc Natl Acad Sci USA 111, E1950–E1959. 24782544 10.1073/pnas.1406161111 PMC4024920

[tjp12942-bib-0016] Miceli F , Vargas E , Bezanilla F & Taglialatela M (2012). Gating currents from Kv7 channels carrying neuronal hyperexcitability mutations in the voltage‐sensing domain. Biophys J 102, 1372–1382. 22455920 10.1016/j.bpj.2012.02.004 PMC3309409

[tjp12942-bib-0017] Musset B , Smith SM , Rajan S , Morgan D , Cherny VV & Decoursey TE (2011). Aspartate 112 is the selectivity filter of the human voltage‐gated proton channel. Nature 480, 273–277. 22020278 10.1038/nature10557 PMC3237871

[tjp12942-bib-0018] Papazian DM , Shao XM , Seoh SA , Mock AF , Huang Y & Wainstock DH (1995). Electrostatic interactions of S4 voltage sensor in Shaker K^+^ channel. Neuron 14, 1293–1301. 7605638 10.1016/0896-6273(95)90276-7

[tjp12942-bib-0019] Pless SA , Galpin JD , Niciforovic AP & Ahern CA (2011). Contributions of counter‐charge in a potassium channel voltage‐sensor domain. Nat Chem Biol 7, 617–623. 21785425 10.1038/nchembio.622 PMC4933587

[tjp12942-bib-0020] Sokolov S , Scheuer T & Catterall WA (2007). Gating pore current in an inherited ion channelopathy. Nature 446, 76–78. 17330043 10.1038/nature05598

[tjp12942-bib-0021] Starace DM & Bezanilla F (2004). A proton pore in a potassium channel voltage sensor reveals a focused electric field. Nature 427, 548–553. 14765197 10.1038/nature02270

[tjp12942-bib-0022] Starace DM , Stefani E & Bezanilla F (1997). Voltage‐dependent proton transport by the voltage sensor of the Shaker K^+^ channel. Neuron 19, 1319–1327. 9427254 10.1016/s0896-6273(00)80422-5

[tjp12942-bib-0023] Struyk AF & Cannon SC (2007). A Na^+^ channel mutation linked to hypokalemic periodic paralysis exposes a proton‐selective gating pore. J Gen Physiol 130, 11–20. 17591984 10.1085/jgp.200709755 PMC2154364

[tjp12942-bib-0024] Tao X , Lee A , Limapichat W , Dougherty DA & MacKinnon R (2010). A gating charge transfer center in voltage sensors. Science 328, 67–73. 20360102 10.1126/science.1185954 PMC2869078

[tjp12942-bib-0025] Tombola F , Pathak MM , Gorostiza P & Isacoff EY (2007). The twisted ion‐permeation pathway of a resting voltage‐sensing domain. Nature 445, 546–549. 17187057 10.1038/nature05396

[tjp12942-bib-0026] Tombola F , Pathak MM & Isacoff EY (2005). Voltage‐sensing arginines in a potassium channel permeate and occlude cation‐selective pores. Neuron 45, 379–388. 15694325 10.1016/j.neuron.2004.12.047

[tjp12942-bib-0027] Tombola F , Ulbrich MH & Isacoff EY (2008). The voltage‐gated proton channel Hv1 has two pores, each controlled by one voltage sensor. Neuron 58, 546–556. 18498736 10.1016/j.neuron.2008.03.026 PMC2430592

[tjp12942-bib-0028] Voets T , Owsianik G , Janssens A , Talavera K & Nilius B (2007). TRPM8 voltage sensor mutants reveal a mechanism for integrating thermal and chemical stimuli. Nat Chem Biol 3, 174–182. 17293875 10.1038/nchembio862

[tjp12942-bib-0029] Vriens J , Held K , Janssens A , Toth BI , Kerselaers S , Nilius B , Vennekens R & Voets T (2014). Opening of an alternative ion permeation pathway in a nociceptor TRP channel. Nat Chem Biol 10, 188–195. 24390427 10.1038/nchembio.1428

[tjp12942-bib-0030] Vriens J , Owsianik G , Hofmann T , Philipp SE , Stab J , Chen X , Benoit M , Xue F , Janssens A , Kerselaers S , Oberwinkler J , Vennekens R , Gudermann T , Nilius B & Voets T (2011). TRPM3 is a nociceptor channel involved in the detection of noxious heat. Neuron 70, 482–494. 21555074 10.1016/j.neuron.2011.02.051

[tjp12942-bib-0031] Vriens J , Owsianik G , Janssens A , Voets T & Nilius B (2007). Determinants of 4α‐phorbol sensitivity in transmembrane domains 3 and 4 of the cation channel TRPV4. J Biol Chem 282, 12796–12803. 17341586 10.1074/jbc.M610485200

[tjp12942-bib-0032] Wagner TF , Loch S , Lambert S , Straub I , Mannebach S , Mathar I , Dufer M , Lis A , Flockerzi V , Philipp SE & Oberwinkler J (2008). Transient receptor potential M3 channels are ionotropic steroid receptors in pancreatic β cells. Nat Cell Biol 10, 1421–1430. 18978782 10.1038/ncb1801

[tjp12942-bib-0033] Wu F , Mi W , Hernandez‐Ochoa EO , Burns DK , Fu Y , Gray HF , Struyk AF , Schneider MF & Cannon SC (2012). A calcium channel mutant mouse model of hypokalemic periodic paralysis. J Clin Invest 122, 4580–4591. 23187123 10.1172/JCI66091 PMC3533564

[tjp12942-bib-0034] Zhao J & Blunck R (2016). The isolated voltage sensing domain of the Shaker potassium channel forms a voltage‐gated cation channel. Elife 5, e18130. 27710769 10.7554/eLife.18130 PMC5092046

